# Identification of New *Halomonas* Strains from Food-related Environments

**DOI:** 10.1264/jsme2.ME21052

**Published:** 2022-03-16

**Authors:** Ayaka Tsuji, Yasuko Takei, Taku Nishimura, Yoshinao Azuma

**Affiliations:** 1 Graduate School of Biology-Oriented Science and Technology, Kindai University, Wakayama, Japan; 2 Energy Technology Laboratories, OSAKA GAS Co., Ltd., Osaka, Japan

**Keywords:** polyhydroxybutyrate, *Halomonas*, genome ana­lysis, ectoine

## Abstract

*Halomonas* species, which are aerobic, alkaliphilic, and moderately halophilic bacteria, produce diverse biochemicals. To identify food-related *Halomonas* strains for bioremediation and the industrial production of biochemicals, 20 strains were isolated from edible seashells, shrimp, and umeboshi (pickled Japanese plum) factory effluents. All isolates were phylogenetically classified into a large clade of *Halomonas* species. Most isolates, which grew in wide pH (6–13) and salt concentration (0–14%) ranges, exhibited the intracellular accumulation of poly(3-hydroxybutyrate) granules. The characteristics of these isolates varied. A020 isolated from umeboshi factory effluents exhibited enhanced stress tolerance and proliferation and comprised two plasmids. IMZ03 and A020 grew to more than 200 OD_600_, while IMZ03 produced 3.5% 3-hydroxybutyrate in inorganic medium supplemented with 10% sucrose. The mucus of TK1-1 cultured on agar medium comprised approximately 64‍ ‍mM of ectoine. Whole-genome sequencing of A020 was performed to elucidate its origin and genomic characteristics. The genome ana­lysis revealed a region exhibiting synteny with a large virus genome isolated from the ocean, but did not identify any predictable pathogenic genes. Therefore, saline foods and related materials may be suitable resources for isolating *Halomonas* strains exhibiting unique, useful, and innocuous features.

Members of the genus *Halomonas* are Gram-negative, aerobic, mesophilic, alkaliphilic, and moderately halophilic bacteria. The first species of this genus to be isolated was *Halomonas elongata* from a solar salt facility (Vreeland *et al.*, 1980). Several *Halomonas* species have since been isolated from natural saline habitats (Quesada *et al.*, 1984), marine animals, such as marine ascidians from the Sea of Japan, Russia (Romanenko *et al.*, 2002), and a sea urchin from the South China Sea (Chen *et al.*, 2009). Pathogenic *Halomonas* sp. CAM2 was identified as an etiological factor for epizootics among scallop larval cultures in Chilean hatcheries (Rojas *et al.*, 2009). *Halomonas taeanensis* in combination with other marine bacteria functions as a marine probiotic and increases the resistance of corals to bleaching (Rosado *et al.*, 2019). *Halomonas* species were also previously identified from salted foods, such as traditional cheeses in Europe (Maoz *et al.*, 2003), jeotgal (Korean fermented seafood) (Yoon *et al.*, 2002), Chinese fermented fish sauce (Jiang *et al.*, 2014), and stinky tofu (Gu *et al.*, 2018). Although *Halomonas* species are moderately halophilic, metagenomic studies identified *Halomonas* in freshwater animals. The abundance of *Halomonas* in the gut microbiome of wild freshwater fish species, such as carp in China, was found to be up to 16% (Liu *et al.*, 2016). Similarly, *Halomonas* comprised up to 10% of the intestinal microbes in laboratory-reared zebrafish (Yang *et al.*, 2017). Furthermore, *Halomonas* bacteria were commonly detected in brine shrimp in the Great Salt Lake, USA (Riddle *et al.*, 2013). Therefore, the major source of *Halomonas* in zebrafish may be brine shrimp, which are routinely used as a food source for laboratory-reared zebrafish.

*Halomonas* species exhibit various biochemical functions, such as denitrification (Berendes *et al.*, 1996) and the degradation of hazardous phenolic compounds (Garcia *et al.*, 2004). A number of *Halomonas* strains have been isolated and characterized from extreme natural and artificial environments, including heavy metal-contaminated lakes (de Souza *et al.*, 2001), industrial sewage (Yoshie *et al.*, 2006), and chemically polluted environments (Orphan *et al.*, 2000). Moreover, *Halomonas* strains produce various biochemicals, such as ectoine (Wohlfarth *et al.*, 1990) and polyhydroxybutyrate (PHB) (Sierra and Gibbons, 1962). Ectoine (1,4,5,6-tetrahydro-2-methyl-4-pyrimidine carboxylic acid), a widely used low-molecular-weight organic osmolyte, was initially identified in the halophilic bacterium *Ectothiorhodospira halochloris* (Galinski *et al.*, 1985). Previous studies reported that ectoine protected cells against various environmental stressors, such as extreme temperatures, high salt concentrations, and ultraviolet radiation (Knapp *et al.*, 1999). PHB, a biodegradable thermoplastic material, is expected to become a substitute for petrochemical plastics (Lee, 1996). The production of ectoine and PHB may be a strategy by which *Halomonas* adapt to low nutrient and high salt (leading to high osmotic pressure) conditions (Anderson and Dawes, 1990). *Halomonas* sp. KM-1 was shown to produce (*R*)-3-hydroxybutyrate (3HB) (Kawata *et al.*, 2012), which is synthesized from PHB through the activity of PHB depolymerase (Jendrossek *et al.*, 1995). The preference of *Halomonas* for hypersaline and alkaline conditions may be attributed to ectoine and monovalent cation proton antiporters. Ectoine is a major compatible organic solute for some halophilic microorganisms to adjust intracellular osmotic pressure (Canovas *et al.*, 1997) and cope with environmental stressors, such as temperatures (Oren, 2002) and reactive oxygen species (Brands *et al.*, 2019). Genomic and experimental ana­lyses have characterized the ectoine metabolic pathways of *H. elongata* (Schwibbert *et al.*, 2011).

In Japan, approximately 70,000 tons of fully ripened *Prunus mume* fruit are pickled annually to prepare umeboshi, which is a highly salty and sour traditional health food. Commercially popular umeboshi is currently produced through the re-pickling of traditional umeboshi in seasoning liquids to decrease its saltness and sourness. The market for umeboshi has been stable for the past three decades because its taste and composition are compatible with Japanese traditional food in addition to the desire of consumers to lead healthy lifestyles (Enomoto *et al.*, 2010; Kono *et al.*, 2018). Industrial seasoning liquid effluents contain approximately 10% saccharides, 8% salt, and 3% citric acid. Therefore, industrial effluents must be diluted by up to 100-fold with water before subjecting them to the activated sludge process in order to decrease their biological oxygen demand (Takatsuji *et al.*, 1999). The establishment of a low-cost and environmentally friendly effluent treatment strategy using halotolerant and harmless microorganisms is needed, similar to the treatment strategy used for olive mill wastewater containing large amounts of salt and organic compounds in the Mediterranean region (Sayadi and Ellouz, 1995).

The present study is the first to isolate *Halomonas* from umeboshi factory effluents and seashells. A genome ana­lysis of umeboshi isolates revealed no pathogenic genes. The present results indicate that bioremediation using safe extremophiles is useful for the treatment of umeboshi factory effluents.

## Materials and Methods

### Sample collection

Umeboshi seasoning effluents were collected from the following factories: JA Kinan (33°46'16.6''N, 135°21'9.1''E) and Iwamoto Foods (33°47'6.5''N 135˚20'37.3''E), Wakayama, Japan. The two factories are located approximately 2‍ ‍km from each other and 4‍ ‍km from the nearest coast. *Turbo sazae* (sazae) and *Ruditapes philippinarum* (Japanese clam) were harvested from fisheries at Kada (34°16'36''N, 135°4'2''E) and Kawaguchi (32°44'50''N, 130°35'12''E) in Wakayama and Kumamoto, Japan, which were approximately 60 and 450‍ ‍km away from the umeboshi factories, respectively (Supplemental Fig. S1). Seashells were alive until used and were edible without pasteurization. Soil (33°47'2.4''N, 135°20'47.6''E), pond sediment (33°47'32.1''N, 135°20'52.1''E), river (33°47'6.5''N, 135°20'37.3''E), and seawater (33°44'31.3''N, 135°19'38.8''E) samples were collected near the umeboshi factories.

### Strains and culture conditions

*Halomonas* sp. KM-1 (FERM BP-10995) was a kind gift from Dr. Kawata, the National Institute of Advanced Industrial Science and Technology.

*Halomonas* species were cultured and isolated in SOT-base medium (pH 9.5, total Na ion concentration corresponding to 2.5% NaCl) supplemented with 3.0% (w/v) sucrose (Kawata and Aiba, 2010). Liquid culturing was performed at 30˚C under agitation. A microbiological ana­lysis was conducted using SOT2-based rich media (pH 10.0). The composition of SOT2-based rich media was as follows (for 1 L): 24.9‍ ‍g sodium glutamate, 0.10‍ ‍g yeast extract, 2.52‍ ‍g NaHCO_3_, 2.00‍ ‍g K_2_HPO_4_, 1.06‍ ‍g Na_2_CO_3_, 1.00‍ ‍g K_2_SO_4_, 0.20‍ ‍g MgSO_4_·7H_2_O, 0.080‍ ‍g Na_2_EDTA·2H_2_O, 0.040‍ ‍g CaCl_2_, and 0.010‍ ‍g FeSO_4_·7H_2_O. Medium was supplemented with sucrose at a final concentration of 3.0% (w/v) unless otherwise specified. Agar concentrations in SOT and SOT2 agar media were 2.0 and 1.5%, respectively. The composition of umeboshi seasoning medium was 1.5% agar and 50% umeboshi effluent, which contained 7.6% NaCl, 2.5% citrate, and 37% sugar (including 8.0% honey), with a pH of 3.0. pH was adjusted to 7.0 with NaOH. The composition of Luria-Bertani (LB) medium was as follows (for 1 L): 10.0‍ ‍g peptone, 5.0‍ ‍g yeast extract, and 10.0‍ ‍g NaCl.

### Isolation of *Halomonas*

Umeboshi effluents were obtained from tanks and comprised a mixture of various umeboshi seasoning liquids used for the re-pickling of traditional umeboshi before processing in activated sludge facilities. Samples were centrifuged at 10,600×*g* at 25°C for 10‍ ‍min. Pellets were resuspended in 1/40 volume of the supernatant and resuspended samples were left undisturbed for 20‍ ‍min. The supernatant was collected and used as a bacterial suspension. The soft parts of seashells (5 g) were soaked in 10‍ ‍mL of phosphate-buffered saline (PBS, pH 7.4) and soaked samples were left undisturbed for 10‍ ‍min. The supernatant was collected and used as a bacterial suspension. Soil and sediments (10 g) were resuspended in 20‍ ‍mL of H_2_O and resuspended samples were left undisturbed for 10‍ ‍min. The supernatant was collected and used as a bacterial suspension.

The bacterial suspension and seawater (100‍ ‍μL) were spread on SOT and SOT2 agar media supplemented with 3.0% sucrose and incubated at 30°C for 3–8 days. Bacterial colonies were subjected to colony isolation twice using the same media.

### 16S rRNA gene sequencing and phylogenetic ana­lysis

DNA fragments encoding the 16S rRNA gene were amplified by a polymerase chain reaction (PCR) with the following primers: 5′-aaactgaagagtttgatcatggc-3′ and 5′-aggtgatccagccgcaggtt-3′. The DNA sequences of the PCR products were elucidated using the same primers as those used for PCR. Genus identification was performed using the Basic Local Alignment Search Tool (BLAST) (Altschul *et al.*, 1990). The multiple sequence alignment and phylogenetic ana­lysis of DNA sequences were performed using ClustalW ver. 2.1 (Larkin *et al.*, 2007) and MEGA X (Kumar *et al.*, 2018), respectively.

### Product ana­lyses

PHA production was examined using Nile red staining (Spiekermann *et al.*, 1999). Briefly, *Halomonas* cells were cultured in SOT liquid medium supplemented with 3% sucrose at 30°C for 1 day. To harvest cells, the bacterial suspension was centrifuged at 21,600×*g* at 4°C for 10‍ ‍min. The cell pellet was resuspended in 4% paraformaldehyde in PBS and incubated at 25°C for 5‍ ‍min. Cells were then washed with PBS and stained with 2.5‍ ‍μg mL^–1^ Nile red in 1% dimethyl sulfoxide/PBS at room temperature for 15‍ ‍min. After washing with PBS, cells were observed under an inverted fluorescence microscope (Eclipse Ti-S; Nikon). The chemical composition of the granules was identified using gas chromatography (Juengert *et al.*, 2018). Sugar concentrations were measured using the phenol-sulfuric acid method (Azuma *et al.*, 2009). The methods for examining 3HB production were previously described (Kawata *et al.*, 2012). Briefly, *Halomonas* cells were incubated at 30°C for 2 days under aerobic conditions with shaking at 200‍ ‍rpm, followed by an incubation at 30°C for 2 days under microaerobic conditions with shaking at 50‍ ‍rpm. The culture was centrifuged and the supernatant was diluted 10-fold with 5‍ ‍mM sulfuric acid and filtered using a disposable 0.2-μm filter. Eluents were subjected to a high-performance liquid chromatography (HPLC) ana­lysis using a HPLC system (Prominence; Shimadzu) equipped with a Aminex®HPX-87H column (Bio-Rad). HPLC conditions were as follows: column temperature, 35°C; mobile phase, 5‍ ‍mM sulfuric acid; flow rate, 0.6‍ ‍mL‍ ‍min^–1^; detector, UV detector; detection wavelength, 210‍ ‍nm.

Ectoine concentrations were measured using HPLC and electrospray ionization time-of-flight mass spectrometry (ESI-TOF MS). Mucus samples (1.0 g) of TK1-1 and other *Halomonas* strains cultured on SOT2 agar medium were suspended in 10‍ ‍mL of 75% acetonitrile. Samples were filtered and subjected to a HPLC ana­lysis using the HPLC system (Prominence, Shimadzu) equipped with an Inertsil Amide column for amino acid separation (GL Science). HPLC conditions were as follows: column temperature, 45°C; mobile phase, 75% acetonitrile; flow rate, 0.6‍ ‍mL‍ ‍min^–1^; detector, UV detector; detection wavelength, 210‍ ‍nm. ESI-TOF MS ana­lyses were performed using a Triple TOF 5600+ system (AB Sciex) equipped with ESI in the positive ion mode. HPLC fractions were applied to TOF MS and ions were monitored in the range of 50 to 500 *m*/*z*.

### Genomic DNA sequencing and comparative ana­lysis

The draft genomic DNA sequences of *Halomonas* isolates were elucidated using a whole-genome shotgun strategy according to a previously described method with minor modifications (Kawai *et al.*, 2015). Briefly, 8.3‍ ‍μg of genomic DNA was extracted from *Halomonas* sp. A020 using the Gentra Puregene Yeast/Bact. Kit (Qiagen). Genomic DNA was fragmented and subjected to PCR-free library preparation using the NEBNext Ultra II DNA Library Prep Kit (New England Biolabs). The library was sequenced using the HiSeq system (Illumina) to obtain 10.5 M pair-end reads (150 bases per read). Short DNA reads were assembled using the CLC Genomics Workbench (Qiagen), which generated 51 contigs (>500 bases and >0.5 copy number) with 219,903 bp of N50 and a total of 3,905,596 bp (coverage 398×). The complete genomic DNA sequence of *Halomonas* sp. A020 was analyzed using a previously described method (Kawai *et al.*, 2015). Unknown DNA sequences in gap regions and uncertain sequences in contigs were elucidated at least once in both strands through the direct sequencing of PCR products. Contig alignment was confirmed through the direct sequencing of genomic DNA using MinION (Oxford Nanopore Technologies). Protein-coding genes and tRNA genes were identified and annotated using an automatic annotation service, DFAST (https://dfast.ddbj.nig.ac.jp) operated by DDBJ (Tanizawa *et al.*, 2018). Unique *Halomonas* genes were analyzed using the BLASTP program (Altschul *et al.*, 1997).

### Data availability

Sequence data in the present study were deposited in DDBJ/EMBL/GenBank under the following accession numbers: *Halomonas* sp. A020 chromosomal sequence, AP022850, AP022851, and AP022852; plasmid sequences, pHA020_1 and pHA020_2.

## Results and Discussion

### Isolation of *Halomonas* species from food and related materials

**Fig. 1.**
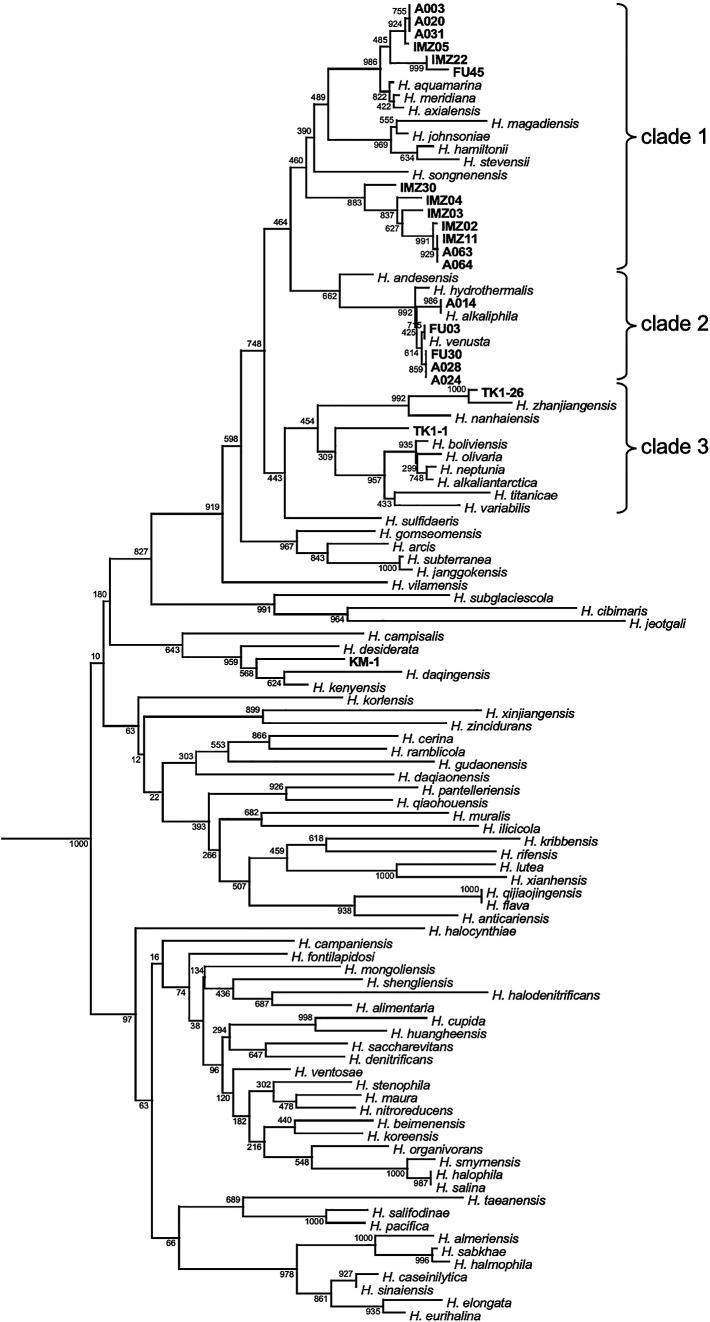
Phylogenetic tree constructed based on 16S rRNA gene sequences of 20 isolates and 82 *Halomonas* type strains. The DNA sequences of the 16S rRNA gene were aligned using ClustalW (ver. 2.1) and MEGA X. The isolation sources of the different strains were as follows: A003-A064, umeboshi effluents; FU03, *Pandalus latirostris* (shrimp); FU30, FU45, TK1-1, and TK1-26, *Turbo sazae* (turban shell); IMZ02-IMZ30, *Ruditapes philippinarum* (Japanese clam). The default parameters of ClustalW and MEGA X were used. *Pseudomonas* and *Vibrio* strains were used to locate the roots of the phylogenetic tree (data not shown).

*Halomonas* bacteria produce diverse and useful biochemicals, such as ectoine and PHB. To identify useful *Halomonas* species that are not harmful to human health and the environment, 20 Japanese commercial salted foods and fresh seafood as well as effluents from three companies manufacturing salted foods were used to isolate *Halomonas*. Based on the DNA sequences of 16s rRNA genes and microbiological ana­lyses, 20 isolates were identified as independent *Halomonas* strains: 4 isolates from *T. sazae* (turban shell), 7 from *R. philippinarum* (Japanese clam), 1 from *Pandalus latirostris* (shrimp), and 8 from effluents of traditional umeboshi manufacturers (Fig. 1). In the present study, *Halomonas* bacteria were not isolated from processed food or environmental samples, such as pickled seafood and seawater.

All 20 isolates were phylogenetically clustered into three clades, which consisted of *Halomonas* species isolated from a number of areas, such as seawater, saline soil, and lakes (Fig. 1). *Halomonas meridiana* assigned to clade 1 was previously isolated from Antarctic saline lakes (James *et al.*, 1990), while *Halomonas venusta* assigned to clade 2 was identified as a marine bacterium (Baumann *et al.*, 1972). While the DNA sequences of the 16S rRNA genes of A003, A020, and A031 from umeboshi effluents were the same, the plasmid numbers in A003, A020, and A031 were one, two, and zero, respectively (Supplemental Fig. S2). Therefore, these isolates were described as independent in the present study.

Approximately 8.4×10^6^ colonies g^–1^ of edible seashell parts was observed on SOT2 agar medium. Of these, 1.0×10^3^ colonies belonged to the genus *Halomonas*. Japanese individuals traditionally consume seashells without cooking. Therefore, these *Halomonas* bacteria may not be the causative agents of food poisoning in humans.

### Microbiological characterization of *Halomonas* isolates

**Fig. 2.**
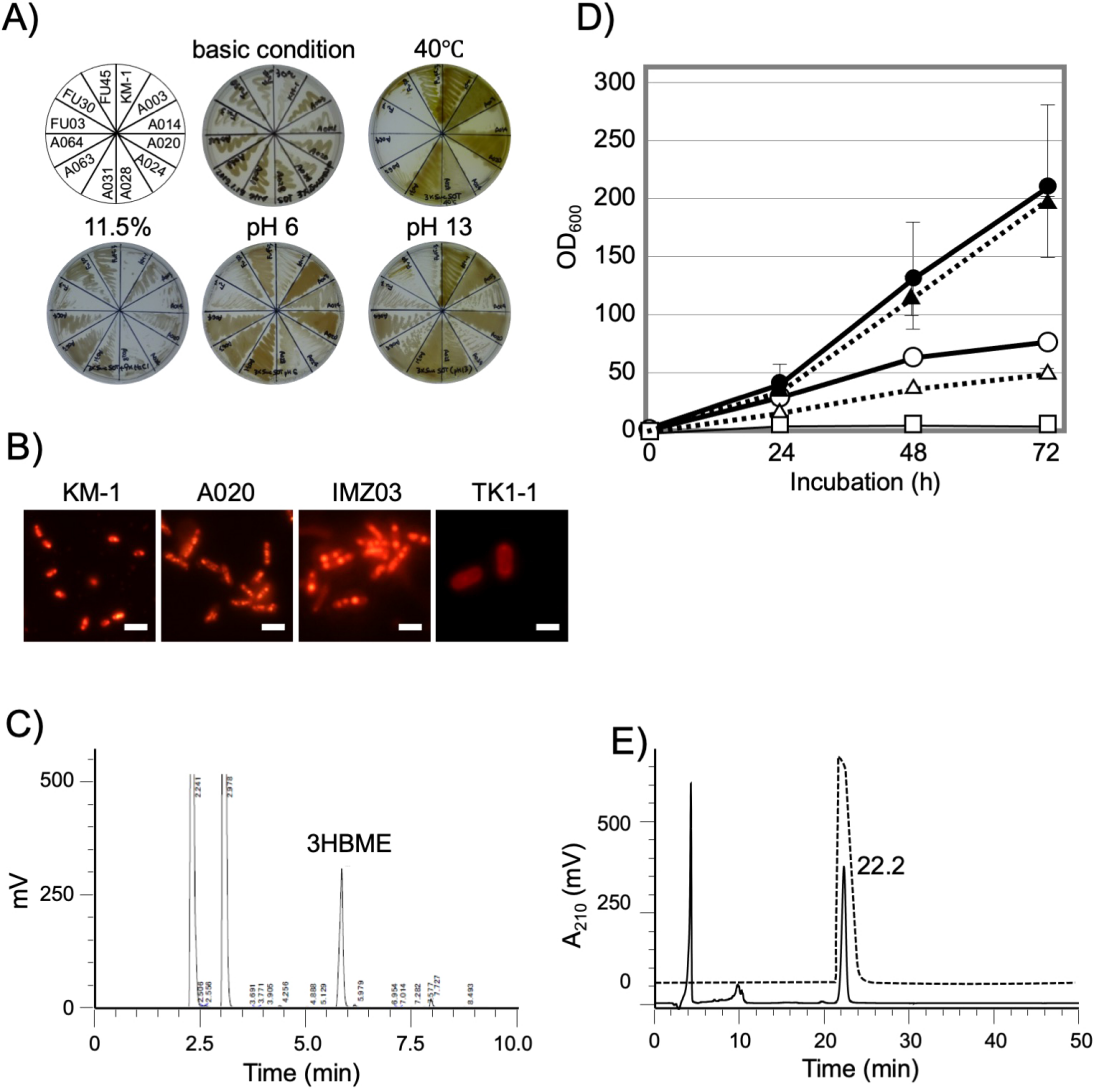
Microbiological characterization of *Halomonas* isolates. A) Eleven* Halomonas* isolates and a control strain (*Halomonas* sp. KM-1) were cultured on SOT agar media (2.5% NaCl, pH 9.5) supplemented with 3% sucrose at 33°C (basic conditions). Modified conditions are indicated on the top of each image. Whole data are shown in Fig. S3 and summarized in Table 1. B) *Halomonas* isolates were cultured in SOT liquid medium supplemented with 3% sucrose at 30°C and 250‍ ‍rpm for 24‍ ‍h. Cells were stained with Nile red. Scale bar: 1‍ ‍μm. Staining intensities in test isolates were compared with those in *Halomonas* sp. KM-1. C) After methanolysis, Nile red-stained intracellular granules were analyzed using gas chromatography (3HBME, 3-hydroxybutyryl methyl ester). D) *Halomonas* sp. IMZ03 (circle) and A020 (triangle/broken line) were cultured in SOT medium supplemented with 10% sucrose (opened) and SOT2 medium supplemented with 10% sucrose (closed) at 30°C under aerobic conditions. All experiments were performed in triplicate. Error bars indicate standard deviations. *E. coli* (square) incubated in LB medium at 37°C under aerobic conditions was used as a control. E) TK1-1 mucus (10% diluted, solid line) and the ectoine standard (broken line on the top) were analyzed using high-performance liquid chromatography.

Isolates were characterized under different temperature, salt concentration, and pH conditions. Three umeboshi isolates (A003, A020, and A031) multiplied under a wide range of salt concentrations (2.5–11.5%), growth temperatures (15–40°C), and pH (6–13) (Fig. 2A, summarized in Table 1 and Supplemental Fig. S3). All umeboshi isolates exhibited rapid growth on umeboshi seasoning agar medium. Other isolates exhibited slow and unstable growth on umeboshi seasoning agar medium, which may be attributed to the low pH of the medium.

Intracellular PHB accumulation is one of the most important features of *Halomonas* species for industrial applications. Most of the isolates exhibited Nile red-stained intracellular granule accumulation. However, the timing of the highest accumulation and the number of intracellular granules varied between different isolates (Fig. 2B and Supplemental Fig. S4). To identify the chemical composition of the granules, partially purified granules from A020 were subjected to methanolysis. A major methanolysis product of A020 granules matched that of the PHB standard (Fig. 2C). Moreover, the significant decrease observed in the level of the methanolysis product under microaerobic conditions was accompanied by the production of 3HB, a PHB monomer. Therefore, the intracellular granules of A020 were confirmed to comprise PHB. The PHB and 3HB productivities of isolates were quantified using gas chromatography and HPLC. IMZ03 isolated from *R. philippinarum* produced 3.5% 3HB in SOT medium supplemented with 10% sucrose, whereas IMZ04 and IMZ30 isolated from *R. philippinarum* and A003 and A020 secreted more than 2.0% of 3HB (Table 1). Most *Halomonas* isolates grew to 100 OD_600_, while A020 and IMZ03 grew to 200 OD_600_ in SOT2 medium supplemented with 10% sucrose (Fig. 2D). The microscopy ana­lysis revealed that significantly high turbidity values were dependent on cell density. The percentages of viable A020 and IMZ03 cells were approximately 50 and 20%, respectively, after a 72-h incubation.

TK1-1 isolated from *T. sazae* produced a large amount of mucus, which contained a compound similar to ectoine upon resolution in an amino acid separation column (Fig. 2E). The HPLC fraction showed signals at an m/z ratio of 143.1 in the first scan mode (MS) and at m/z ratios of 68.0 and 97.1 in the second scan (MS/MS) (Supplemental Fig. S5). Since these signals matched ectoine (Fernandes *et al.*, 2018), TK1-1 mucus was confirmed to contain ectoine. HPLC and MS ana­lyses revealed that the concentration of ectoine in TK1-1 mucus was 64‍ ‍mM (9.1‍ ‍g L^–1^). TK1-1 appeared to more efficiently produce ectoine without optimization than *H. elongata* DSM2581 (Fatollahi *et al.*, 2021).

### Whole-genome ana­lysis

**Fig. 3.**
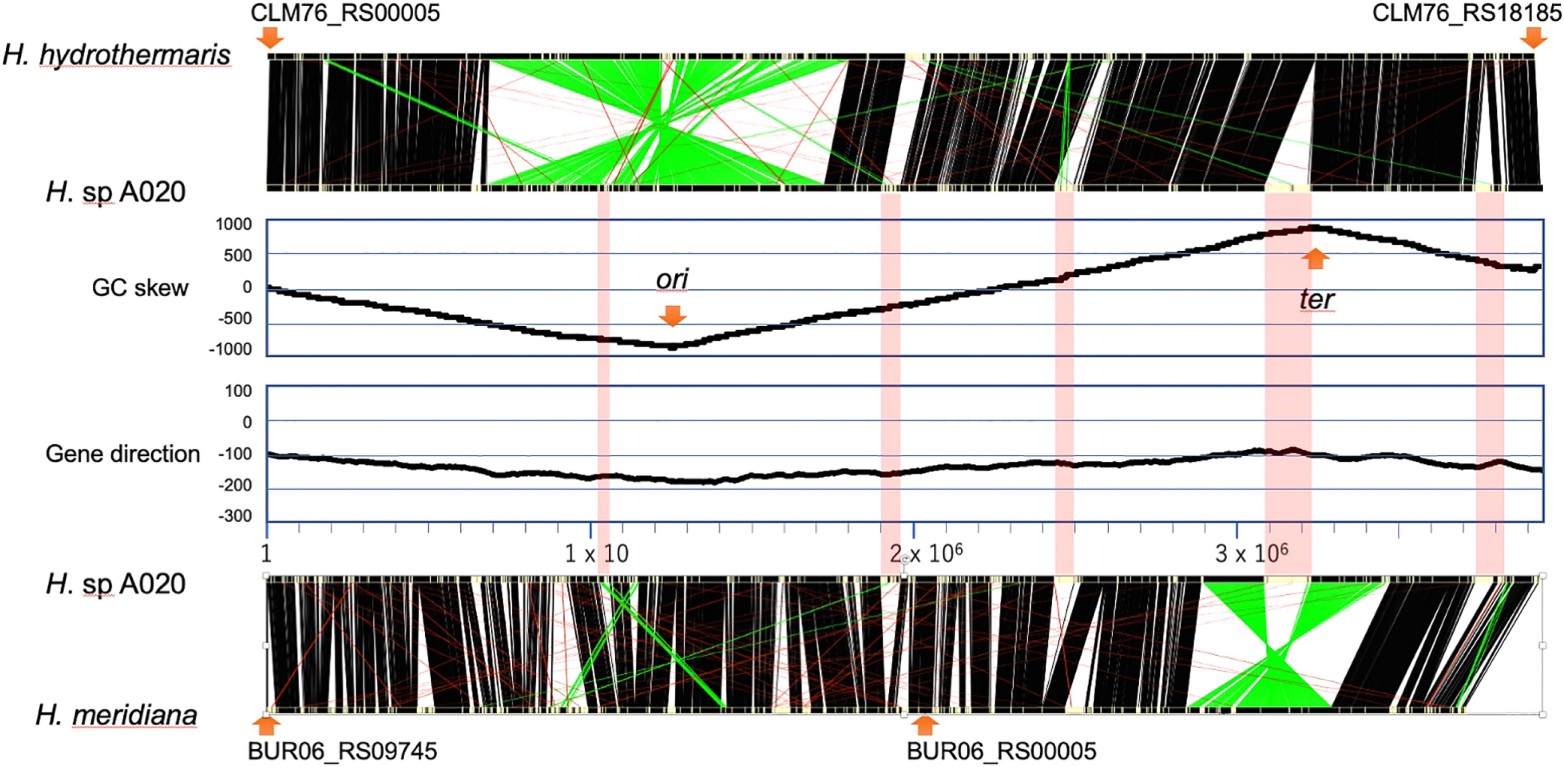
Analysis of the *Halomonas* sp. A020 genome. The positions of the orthologs, which were defined based on a Basic Local Alignment Search Tool (BLAST) bit score >50 and reciprocal best hits, between the genomes of *Halomonas* sp. A020, *Halomonas hydrothermalis* (Bharadwaj Sv *et al.*, 2015), *Halomonas* sp. A020, and *Halomonas meridiana* (Meyer *et al.*, 2015) are indicated at the top and bottom. Black, red, and green lines connect orthologs, which comprise the majority of syntenic genes, syntenic and translocated genes, and translocated genes, respectively. The origin (*ori*) and termination regions of replication (*ter*) were identified based on the GC skew (second top) and cumulus of the transcription directions of genes (third) (Grigoriev, 1998).

Compared with other isolates, *Halomonas* sp. A020 exhibited wider growth ranges of pH (6–13), temperatures (up to 40°C), and salt concentrations (0–12%), and higher PHB and 3HB productivities. Moreover, there were two plasmids in the A020 genome (Supplemental Fig. S2). The whole genomic DNA sequence of A020 was elucidated using a whole-genome shotgun strategy. A020 genomic DNA comprised a circular chromosome (3,957,139 bases) and two plasmids (pHA020_1, 5,809 bases and pHA020_2, 1,571 bases). The DFAST ana­lysis identified six copies of the rRNA gene, 61 tRNA genes, and 3,662 protein-coding genes in the A020 chromosome. The pHA020_1 and pHA020_2 plasmids encoded nine and two protein-coding genes, respectively. GC skew and gene direction ana­lyses indicated the approximate locations of chromosomal *ori* and *ter* (Fig. 3) (Grigoriev, 1998). The comparative ana­lysis of gene locations between A020, *Halomonas hydrothermalis* (Bharadwaj Sv *et al.*, 2015), and *H. meridiana* (Meyer *et al.*, 2015) revealed genome translocation in each case at positions symmetric to the axes of *ori* and *ter*. Among the 3,673 protein-coding genes, 3,177 were well conserved (e value <10^–5^) among *Halomonas* strains, 369 were conserved among certain *Halomonas* and other bacterial strains, and 127 did not exhibit similarities in databases.

**Fig. 4.**
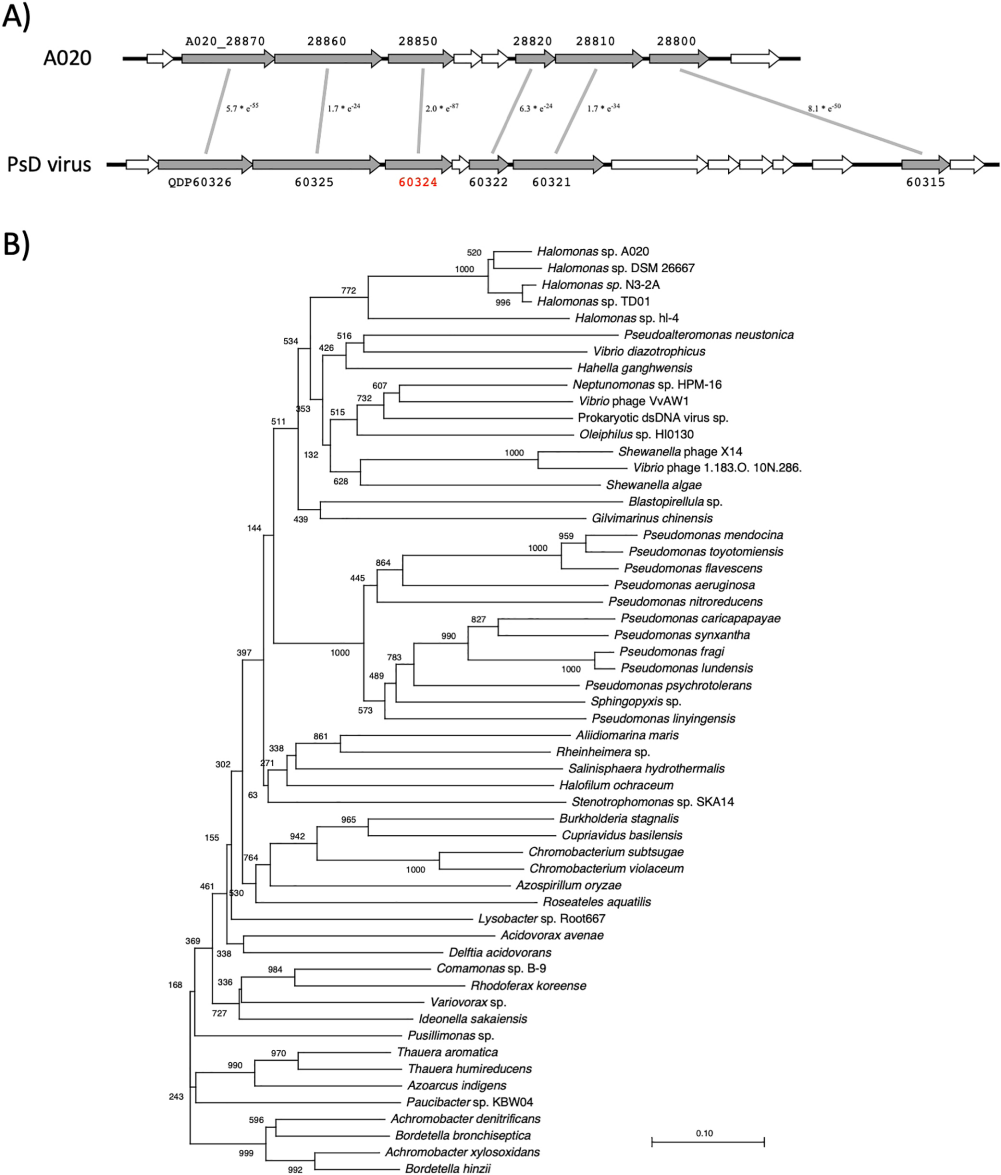
A region in the A020 genome exhibited synteny with prokaryotic double-stranded DNA virus sp. genes. A) Six genes (A020_28870–28800, shown in gray) in the low similarity 94-kbp region adjacent to *ter* in the A020 genome exhibited synteny with prokaryotic double-stranded DNA virus sp. genes (QDP60326–60315) (Roux *et al.*, 2016). B) The A020_28850 gene was conserved among *Vibrio* phages and *Gammaproteobacteria*, including *Halomonas* and *Pseudomonas*, strains.

A020 genomic DNA comprised five phage or transposon insertion regions. The size of the largest insertion region was 94‍ ‍kb, which comprised 126 genes adjacent to *ter* (Fig. 3). A part of this region exhibited synteny with the genomic region of a large virus isolated from the ocean (Fig. 4A) (Roux *et al.*, 2016). This indicated that A020 isolated from the umeboshi factory originated from the ocean. Most of the genes in the synteny region were annotated as hypothetical genes. However, one gene (A020_28850) was conserved among the members of *Gammaproteobacteria*, including *Halomonas*, *Pseudomonas* strains, and *Vibrio* phages (Fig. 4B).

Analyses using the Kyoto Encyclopedia of Genes and Genomes metabolic pathway revealed all genes involved in PHB metabolism in the A020 genome and these genes were conserved among phylogenetically distant strains, such as *H. elongata*, *H. hydrothermalis* Y2, and KM-1. PHB metabolism is a fundamental feature of *Halomonas* bacteria; however, the conditions for polymerization and depolymerization varied between different strains. A unique gene (A020_36530) encoded a large membrane-bound protein (6,022 amino acids), which comprised 20 repeats of a repetitive sequence with 227 amino acids in the N-terminal region and a type I secretion C-terminal target domain (COG2931) in the C-terminal region. The A020_36530 gene was conserved among various *Gammaproteobacteria* strains, particularly *Pseudomonas* and some *Halomonas* strains. However, the number of repetitive sequences varied between the strains. The functions of this protein are unknown.

## Conclusions

The present study is the first to isolate 20 *Halomonas* strains from edible seafood harvested from Japanese seashores and umeboshi factory effluents. Approximately 10^3^ live *Halomonas* bacteria were detected in 1‍ ‍g of seafood. Seafood is traditionally consumed without boiling or baking in Japan, which suggests that *Halomonas* is not a causative agent of food poisoning in humans. A genome ana­lysis of A020 isolates did not reveal any predictable pathogenic genes. Umeboshi isolates are potential candidates for the development of a safe and low-cost bioremediation system for effluents containing high concentrations of salt and organic compounds, including umeboshi factory effluents.

Eight umeboshi isolates were phylogenetically classified into a large clade with other seafood isolates. The genome of the umeboshi isolate A020 comprised a region that exhibited synteny with that of a marine phage. Activated sludge processing facilities for umeboshi factory effluents were located a few kilometers away from the nearest coast. Therefore, the origin of umeboshi isolates may be a nearby ocean (González-Martín *et al.*, 2021). Marine organisms are some of the largest reservoirs of viruses, which may contribute to marine microbial and nutrient homeostasis and gene transfer among marine organisms (Bergh *et al.*, 1989). The syntenic region between the genome of umeboshi isolate A020 and a marine phage genome provides insights into the occurrence of these events.

## Citation

Tsuji, A., Takei, Y., Nishimura, T., and Azuma, Y. (2022) Identification of New *Halomonas* Strains from Food-related Environments. *Microbes Environ ***37**: ME21052.

https://doi.org/10.1264/jsme2.ME21052

## Supplementary Material

Supplementary Material

## Figures and Tables

**Table 1.** Microbiological characteristics

Strains		KM-1	A003	A014	A020	A024	A028	A031	A063	A064	FU3	FU30	FU45	IMZ02	IMZ03	IMZ04	IMZ05	IMZ11	IMZ22	IMZ30	TK1-1	TK1-26
Cell length (μm)		1	1.2	1.8	1.6	1.4	1.7	1.3	1.5	1.8	1.3	1.3	1.3	1.5	1.3	1.5	1.3	1.8	1.3	1.8	1.6	1.7
Production	ectoine	–	–	–	–	–	–	–	–	–	–	–	–	–	–	–	–	–	–	–	+	–
	PHB (g L^–1^)^b^	64.9	6.0	nd^a^	15.6	nd	nd	10.4	nd	nd	nd	nd	nd	19.0	35.4	28.0	18.6	21.7	18.9	12.5	nd	nd
	3HB (g L^–1^)^b^	53.0	14.7	nd	25.6	nd	nd	23.2	nd	nd	nd	nd	nd	3.2	34.7	24.6	10.5	10.8	12.8	26.6	nd	nd
Umeboshi medium		–	+	+	+	+	+	+	+	+	+	+	+	nd	nd	nd	nd	nd	nd	nd	nd	nd
salt tolerance	+3% NaCl	+	+	+	+	+	+	+	+	+	+	+	+	+	+	+	+	+	+	+	+	+
	+6% NaCl	+	+	+	+	+	±	+	+	+	+	+	+	nd	nd	nd	nd	nd	nd	nd	+	+
	+9% NaCl	+	+	–	+	+	±	+	+	+	+	+	+	nd	nd	nd	nd	nd	nd	nd	+	+
	+12% NaCl	–	+	–	+	±	±	+	+	+	±	+	+	nd	nd	nd	nd	nd	nd	nd	+	+
	+15% NaCl	–	±	–	–	–	–	±	±	±	–	–	–	nd	nd	nd	nd	nd	nd	nd	nd	nd
thermo-tolerance	15°C	–	nd	nd	+	nd	nd	nd	nd	nd	nd	nd	nd	nd	nd	nd	nd	nd	nd	nd	+	+
	20°C	–	nd	nd	+	nd	nd	nd	nd	nd	nd	nd	nd	nd	nd	nd	nd	nd	nd	nd	+	+
	25°C	±	nd	nd	+	nd	nd	nd	nd	nd	nd	nd	nd	nd	nd	nd	nd	nd	nd	nd	+	+
	30°C	±	+	±	+	±	+	±	+	+	±	+	+	+	+	+	+	+	+	+	+	+
	33°C–34°C	+	+	+	+	+	+	+	+	+	+	+	+	+	+	+	+	+	+	+	+	–
	36°C–37°C	+	+	+	+	+	+	+	+	+	+	+	+	–	–	–	+	±	–	–	+	–
	39°C–40°C	+	+	–	+	–	+	+	±	–	–	±	+	–	–	–	+	–	–	–	–	–
	42°C	+	+	–	–	–	+	–	–	–	–	–	–	–	–	–	–	–	–	–	–	–
pH	13	+	+	+	+	+	+	+	+	+	+	+	+	nd	nd	nd	nd	nd	nd	nd	nd	nd
	12	+	+	+	+	+	+	+	+	+	+	+	+	nd	nd	nd	nd	nd	nd	nd	nd	nd
	11	+	+	+	+	+	+	+	+	+	+	+	+	nd	nd	nd	nd	nd	nd	nd	nd	nd
	10	+	+	+	+	+	+	+	+	+	+	+	+	nd	nd	nd	nd	nd	nd	nd	nd	nd
	8	+	+	+	+	+	+	+	+	+	+	+	+	nd	nd	nd	nd	nd	nd	nd	nd	nd
	7	–	+	+	+	–	+	+	+	+	±	+	+	nd	nd	nd	nd	nd	nd	nd	nd	nd
	6	–	+	+	+	–	+	+	+	+	–	–	+	nd	nd	nd	nd	nd	nd	nd	nd	nd
	5	–	–	–	–	–	–	–	–	–	–	–	–	nd	nd	nd	nd	nd	nd	nd	nd	nd

^a^ nd, not determined; PHB, polyhydroxybutyrate; 3HB, 3-hydroxybutyrate ^b^ PHB and 3HB (g L^–1^) data were the highest among the experiments performed using SOT2 medium supplemented with 10% sucrose.
