# Supplementary material for: Identification of New Halomonas Strains from Food-related Environments
Source: Microbes Environ. 2022 Mar 16;37(1):ME21052. doi: 10.1264/jsme2.ME21052 (PMC8958296; doi:10.1264/jsme2.ME21052)
Supplement: Supplementary file 1 — Supplementary Material [file 37_21052_s1.pdf]

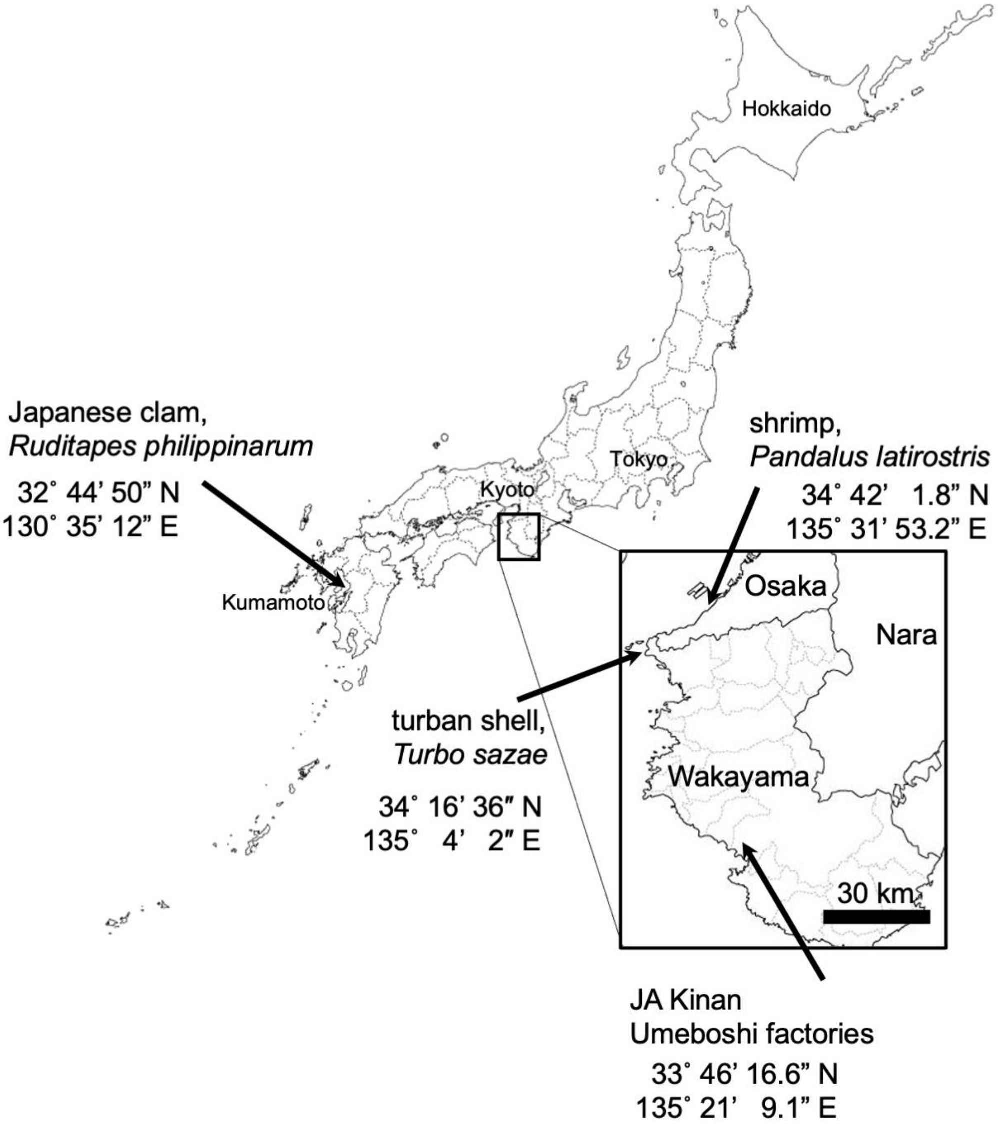

Supplemental Fig. S1. Location of samples prepared for *Halomonas* isolation in Japan.
Turbo sazae and umeboshi effluents were obtained in Wakayama. *Pandalus latirostris* and *Ruditapes philippinarum* were from Osaka and Kumamoto prefecture, respectively.

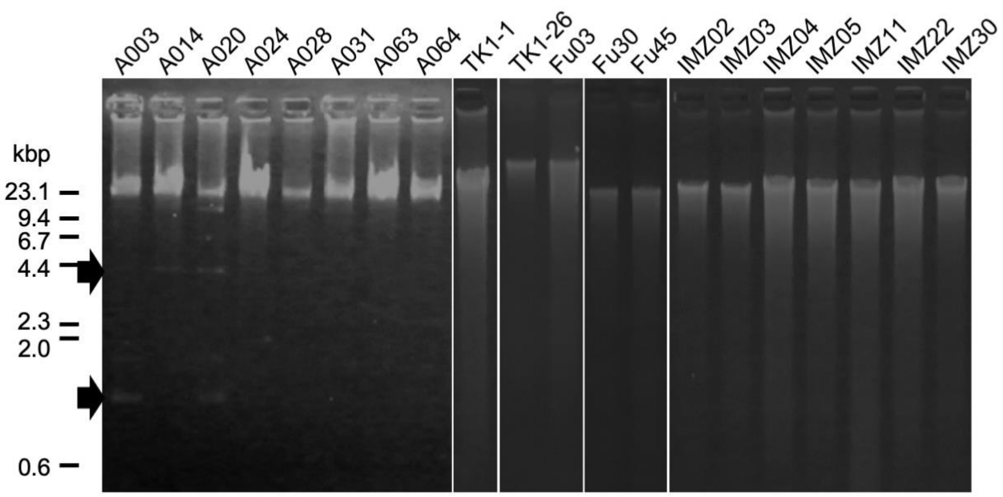

Supplemental Fig. S2. Plasmid DNAs in *Halomonas* sp A020.

Approximately 5 μg of total genomic DNAs extracted from the 20 isolates were analyzed using agarose gel electrophoresis. Positions of plasmid DNAs, which were found in A003, A014 and A020, are indicated with two black arrows. DNA sizes are shown on left.

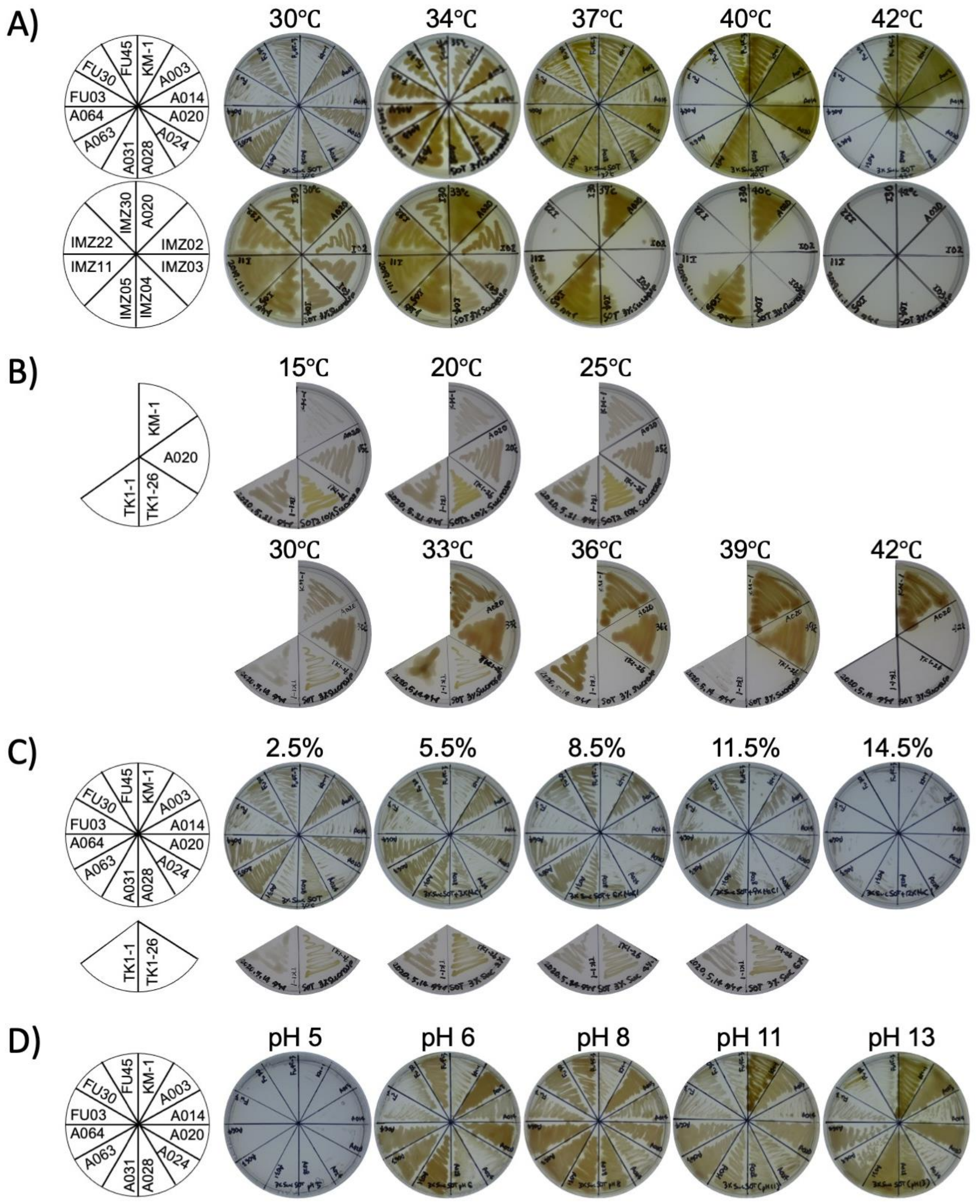

Supplemental Fig. S3. Growth ranges of *Halomonas* isolates.

A) Eighteen isolates were incubated at different temperatures from 30˚C to 42˚C for 3 days on SOT (2.5% NaCl, pH9.5) +3% sucrose. Incubation temperatures were shown on the top of plates. *Halomonas* sp. KM-1 was used as a control. Positions of the strains were shown in the right circles. B) Three isolates were incubated at different temperatures from 15˚C to 42˚C for 3 days on SOT (2.5% NaCl, pH9.5) +3% sucrose.

C) Thirteen strains were tested on different concentrations of NaCl at 30˚C for 3 days. Final concentrations of NaCl in SOT+3% sucrose was shown on the top of plate. D) Eleven strains were tested on media with different pH at 30˚C for 3 days. The pH 5-8 and pH 10-13 were adjusted by addition of citric acid and NaOH, respectively.

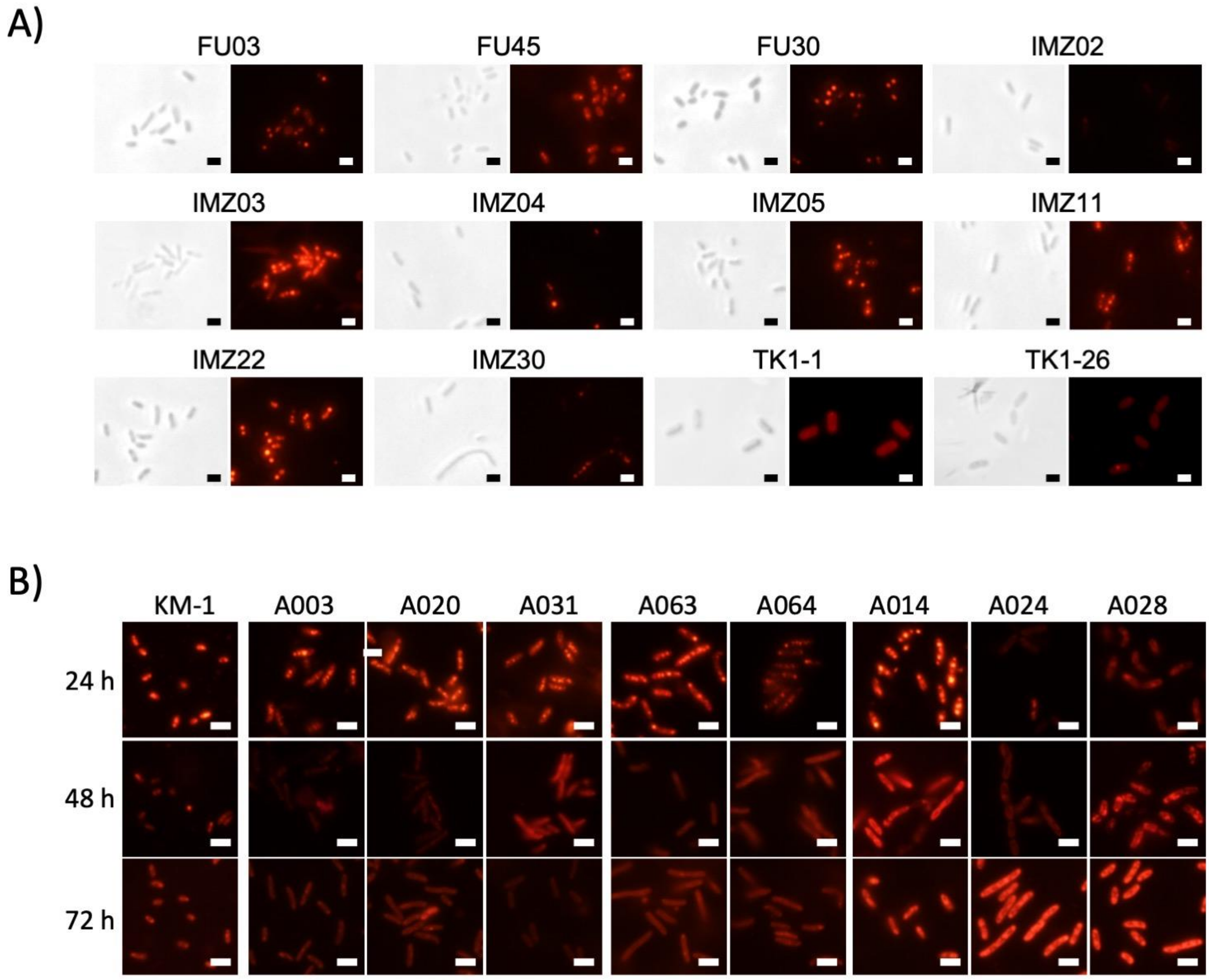

Supplemental Fig. S4. Nile red staining of *Halomonas* isolates.

Halomonas isolates were grown in SOT liquid medium with 3% sucrose at 30˚C and 250 rpm for 24 hours (panel A) or indicated hours (panel B). The cells were stained using Nile red (each right photo in panel A and all in panel B). Bars in white and black indicate 1 µm. *Halomonas* sp. KM-1 was used to compare the staining stutas.

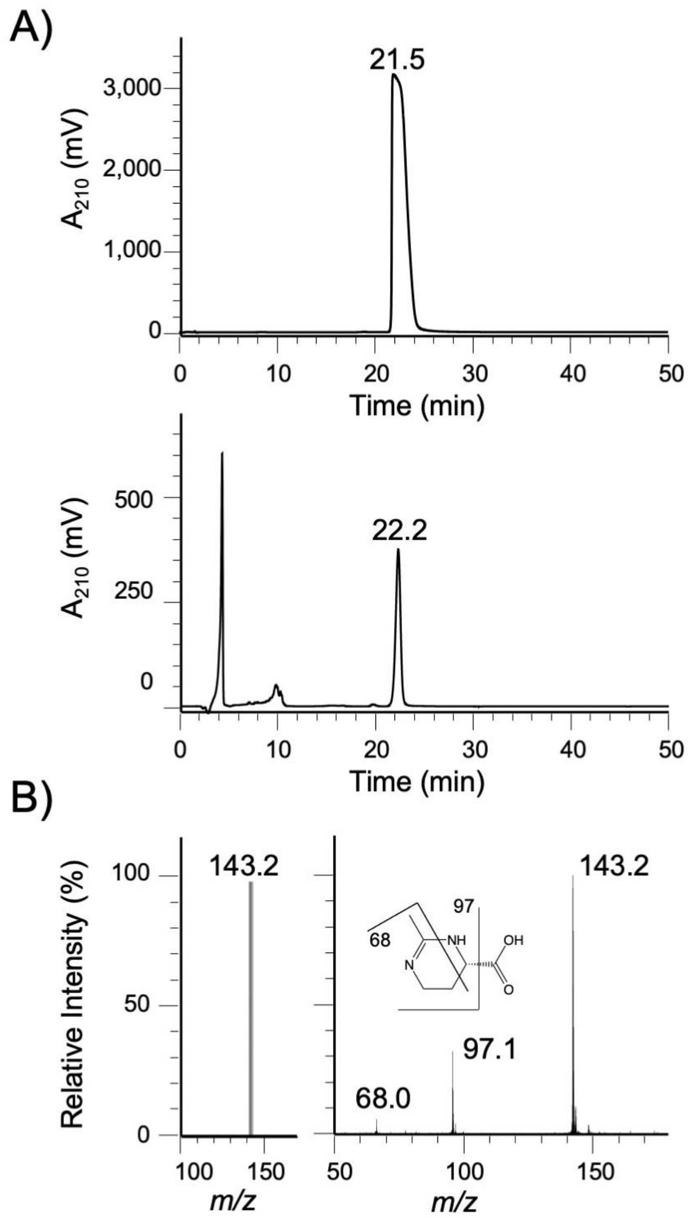

Supplemental Fig. S5. Ectoin analysis using HPLC and MS/MS.

A) The ectoin standard (100 mM, top panel) and TK1-1 mucus (10% diluted, bottom) were analyzed and detected using HPLC with Inertsil Amide column and a UV detector at 210 nm. B) The fruction at 22.2 min of TK1-1 mucus from HPLC contained a molecule with 143.1 m/z in the first scan, and it was found to turn to 68.0 and 97.1 in the second one, which matched with the results published by Fenizia et al. (2020).
